# Calibrating TrueBeam jaws by considering collimator walkout to improve the dose uniformity at abutting field junctions

**DOI:** 10.1002/acm2.12586

**Published:** 2019-04-08

**Authors:** Robert A. Corns, Yingli Zhao

**Affiliations:** ^1^ Department of Radiation Oncology Brody School of Medicine East Carolina University Greenville NC USA; ^2^ Medical Physics Fraser Valley Centre British Columbia Cancer Agency Surrey BC Canada

**Keywords:** calibration, collimator, jaw, junction dose, TrueBeam, walkout

## Abstract

Jaw positions on a linear accelerator are calibrated to have accurate field size values over the range of jaw positions and to have excellent junctions when matching fields. It is sufficient to have field size accuracy on the order of a millimeter for most clinical applications but good junctions require submillimeter precision and accuracy in the jaw positioning. Presented is a method to measure collimator walkout with the MV imager and a mathematical model to determine an optimal origin for calibrating jaws on the TrueBeam accelerator. The calibration procedure uses the jaw position encoders which are sufficiently accurate and precise enough to achieve a homogeneous junction dose for abutting fields.

## 1 INTRODUCTION

Accurate jaw calibration of medical linear accelerators (Linac) with a precision on the order of a millimeter is a requirement in modern quality assurance protocols.^1^, ^2^ Accurate field sizes and a homogeneous junction dose benefit clinical applications such as mono‐isocentric half‐beam block breast cancer or head and neck cancer treatment with field matching.^3^, ^4^ Junction requirements in the Canadian Partnership for Quality Radiotherapy (CPQR) protocol^2^ specify variances in terms of the dosimetry – 5% tolerance and 10% action levels for the dose peak/valley across the junction of the abutting fields – but the reality is this constrains the jaw position's accuracy and precision to be less than half a millimeter.^5^, ^6^ To add to the complexity one often wishes to match jaws that have a 90° collimator rotation between them and the collimator walkout becomes a serious consideration.^5^, ^7^, ^8^, ^9^

The TrueBeam accelerator (Varian Medical Systems, Palo Alto, CA, USA) has a seemingly simple procedure to calibrate the jaw positions which establishes jaw positions with the control system. The procedure is called System Calibrate or Readout Calibration (Using Field Light) for Varian TrueBeam.^10^ Using the Varian IEC scale, the procedure requires user to move the jaw to (a) 1 cm and capture this position as a calibration point; then (b) to 19 cm and capture this position as a second calibration point; and finally (c) verify the calibration by automatically moving to the jaw to its midrange of motion. This midrange position is 5 cm for a Y‐jaw, which can move from −10 to 20 cm and is 9 cm for an X‐jaw, which can move from −2 to 20 cm.

There are a number of technical challenges associated with this task. These are rooted in deciding on where the origin is and moving the jaws to 1, 19, and either 5 or 9 cm from this origin.^10^ The choice of origin must have a number of desirable properties. First, each jaw has its own origin that is independent of the other jaws and ideally all four origins coincide spatially. Second, the origin needs to be centrally located so that the symmetric jaw field sizes are actually symmetric about this point. Third, the origin needs to be on the collimator axis of rotation so that the jaw matching will be less affected by collimator walkout.^9^

If matching the X1 jaw to the X2 jaw and the Y1 jaw to the Y2 jaw were considered alone then the origin could be moved anywhere within a small margin of the collimator axis of rotation and produce an acceptable junction.^11^, ^12^ The jaws in this situation are geometrically matched for good dosimetry and close enough to the axis of rotation so as to maintain accuracy and precision over the entire range of motion. Matching X‐Y jaws requires a 90° collimator rotation and the collimator's walkout will place more restrictions on the origin choice.^9^

## 2 METHODS

### 2.A Theory

**Figure 1:**
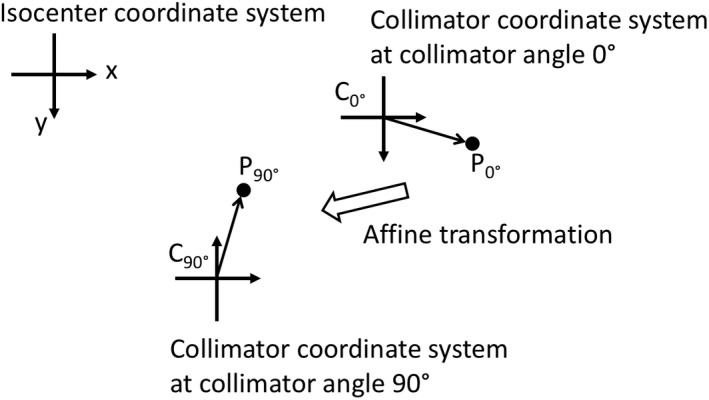
Two coordinate systems are used to characterize the collimator rotations. The isocenter coordinate system is fixed in space on the plane of the isocenter. The collimator coordinate system describes points that are fixed to the collimator, such as a point on a jaw. As the collimator rotates a point *P* will stay fixed relative to the collimator but move in the isocenter coordinates. An affine mapping describes the changes to *P* in position and orientation in the isocenter coordinate system as $P_{0^{\circ }}\mapsto P_{90^{\circ }}$.

When matching jaws with a 90° collimator rotation with minimal effect by the collimator walkout, consider two Cartesian coordinate systems. One is fixed on the isocenter plane with its axes parallel to the in‐line and cross‐line directions. When the MV imaging panel is set on the isocenter plane, its pixel columns and rows represent coordinates in this system. The second coordinate system rotates with the collimator and is also set on to the isocenter plane. The same scaling for distance is used for both coordinate systems. A point fixed to the collimator will maintain its position relative to the collimator coordinate system but moves in the isocenter coordinate system. An example of this would be the position of a shadow cast by a ball bearing fixed on a block tray. These translations and rotations in the isocenter coordinate system, as illustrated in Fig. 1, can be defined by an affine transformation.

When matching X jaws to Y jaws the 0°–90° or 0°–270° collimator angle pairs are typically used and the discussion's focus is restricted to these angles. Starting with an arbitrary origin in the collimator coordinate system, the properties of the affine transformation are used to pick the best location for the jaw origin. Denote the collimator coordinate's origin location *C* in the isocenter coordinate system by *C*_0°_, *C*_90°,_ and *C*_270°_ for collimator angles 0°, 90°, and 270° respectively. They are related to each other by shifts and rotations as

(1) $$C_{90^{\circ }}=C_{0^{\circ }}+\left(s,t\right)$$

(2) $$C_{270^{\circ }}=C_{0^{\circ }}+\left(u,v\right)$$

**Figure 2:**
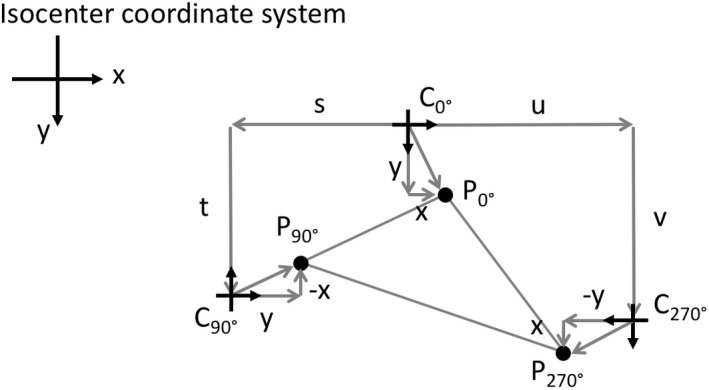
The collimator coordinate system for collimator angles 0°, 90° and 270° and the affine mappings in the isocenter coordinate system: *C*_0°_ ↦ *C*_90°_; *C*_0°_ ↦ *C*_270°_; *P*_0°_ ↦ *P*_90°_; *P*_0°_ ↦ *P*_270°_.

A point of interest *P* that is fixed relative to the collimator has positions *P*_0°_, *P*_90°_, and *P*_270°_ in the isocenter coordinate system. Figure 2 illustrates the relationship between the P's and C's. The points *P*_0°_, *P*_90°_, and *P*_270°_ are related to their respective collimator origins in the isocenter coordinate system by

(3) $$P_{0^{\circ }}=C_{0^{\circ }}+\left(x,y\right)$$

(4) $$P_{90^{\circ }}=C_{90^{\circ }}+\left(y,-x\right)$$

(5) $$P_{270^{\circ }}=C_{270^{\circ }}+\left(-y,x\right)$$

The translational aspect of the affine transformation is contained in the shifts (s, t) and (u, v) while the rotational aspect is describe by $\left(x,y\right)\mapsto \left(y,-x\right)$ and $\left(x,y\right)\mapsto \left(-y,x\right)$ for the 90° and 270° collimator rotations respectively. Note that *x*,* y*,* s*,* t*,* u,* and *v* are all signed quantities and that the positive y direction is downwards on the page in Fig. 2. The (*x*,* y*) direction orientation was chosen to correspond to the direction of increasing pixel numbering on the MV imager.

The point *P* should be chosen to make an ideal origin, which is defined as a point that maps onto itself under both affine transformations *P*_0°_ = *P*_90°_ = *P*_270°_. The reality is these are not all equal due to movement of collimator axis of rotation and there will be choices to make as to what constitutes the best origin. Three different strategies are presented that result in different choices for the location of the origin but this is not an exhaustive list.

#### 2.A.1 Strategy 1: A perfect 0°–90° junction by compromising the 0°–270° junction

Clinically a field with a 270° collimator rotation can be reproduced by a field with a 90° collimator rotation. Hence one could consider only the 0°–90° junction. There is a point that maps to itself under the *C*_0°_ ↦ *C*_90°_ affine transformation that is located at *C*_*0°*_ + (*x*,* y*) with

(6) $$x=\frac{s+t}{2}$$

(7) $$y=\frac{t-s}{2}$$

**Figure 3:**
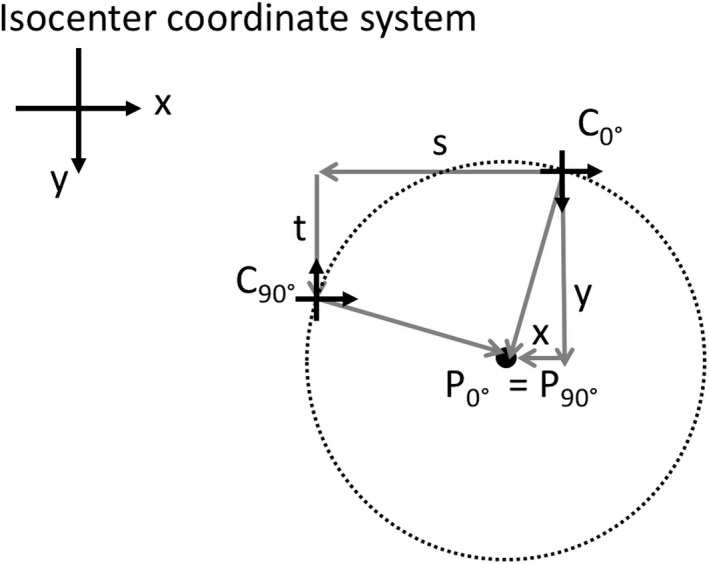
There is a fixed point to the affine transformation *C*_0°_ ↦ *C*_90°_ defined by the location *x* = (*s + t*)/2 and *y* = (*t* – *s*)/2. This point is the center of the circle that traces through points *C*_0°_ and *C*_90°_ and it represents the ideal choice for the jaw origin because the X jaw will geometrically match the Y jaw when each is set to 0 and the collimator is rotated from 0° to 90°.

Physically this point represents the center of a circle that traces through C_0°_ and C_90°_, as shown in Fig. 3.

**Figure 4:**
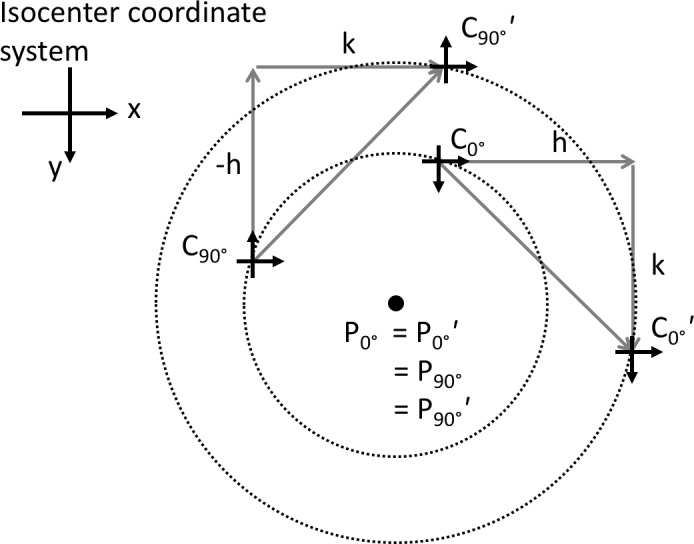
The fixed point to the affine transformation *C*_0°_ ↦ *C*_90°_ is not dependent on the choice *C*_0°_. Any other choice of *C*_0°_ *'* must follow the same affine transformation and both choices define concentric circles whose center is the ideal choice for the origin.

This choice of origin is unique in that does not depend on the choice of the collimator coordinate system's origin. To see this, note that any other choice of the collimator coordinate's origin must necessarily follow the same affine mapping as the original choice of coordinate systems. Consider a new coordinate ${C_{0^{\circ }}}^{\prime }$ shifted by (*h*,* k*) relative to *C*_0°_. Figure 4 illustrates their relation.

(8) $${C_{0^{\circ }}}^{\prime }=C_{0^{\circ }}+\left(h,k\right)$$

The affine transformation law implies the coordinate system ${C_{90^{\circ }}}^{\prime }$ will be at

(9) $${C_{90^{\circ }}}^{\prime }=C_{90^{\circ }}+\left(k,-h\right)$$

The primed coordinate system moves on a circle that is concentric with the one defined by the original unprimed coordinate system. The origin choice is the center of the circle and this is the same for the primed and unprimed collimator coordinates.

#### 2.A.2 Strategy 2: Compromise the perfect 0°–90° junction to improve the 0°–270° junction

If the 0°–270° junction is used clinically, the solution from the first strategy will put the point *P*_270°_ at

(10) $$P_{270^{\circ }}=C_{0^{\circ }}+\left(u-\frac{t-s}{2},v+\frac{s+t}{2}\right)$$

and the difference between *P*_0°_ and *P*_270°_ is

(11) $$P_{270^{\circ }}-P_{0^{\circ }}=\left(u-t,v+s\right)$$

Clearly if *u = t* and *v = –s* then there is a perfect junction between 0° and 270° as well. In practice, these points will differ. If the one or both of the differences |u−t| and |v+s| is larger than some clinically acceptable value then this difference can be improved at the expense of the 0°–90° junction. Consider picking a point *P*_0°_ at the location

(12) $$x=\frac{s+t}{2}+\varepsilon$$

(13) $$y=\frac{t-s}{2}+\delta$$

where the *ε* and *δ* are perturbations from Strategy 1's ideal location. Then *P*_90°_ – *P*_0°_ and *P*_270°_ – *P*_0°_ are

(14) $$P_{90^{\circ }}-P_{0^{\circ }}=\left(\delta -\varepsilon ,-\delta -\varepsilon \right)$$

(15) $$P_{270^{\circ }}-P_{0^{\circ }}=\left(u-t-\delta -\varepsilon ,v+s+\varepsilon -\delta \right)$$

Let f and g represent two numbers to be used for scaling purposes and define

(16) $$\delta +\varepsilon =f\left(u-t\right)$$

(17) $$\delta -\varepsilon =g\left(v+s\right)$$

The two differences *P*_90°_–*P*_0°_ and *P*_270°_ – *P*_0°_ become

(18) $$P_{90^{\circ }}-P_{0^{\circ }}=\left(g\left(v+s\right),-f\left(u-t\right)\right)$$

(19) $$P_{270^{\circ }}-P_{0^{\circ }}=\left(\left(1-f\right)\left(u-t\right),\left(1-g\right)\left(v+s\right)\right)$$

The gap sizes are controlled by picking values for f and g. For example by picking each to be &frac12; then the differences are

(20) $$P_{90^{\circ }}-P_{0^{\circ }}=\left(\frac{v+s}{2},-\frac{u-t}{2}\right)$$

(21) $$P_{270^{\circ }}-P_{0^{\circ }}=\left(\frac{u-t}{2},\frac{v+s}{2}\right)$$

Comparing this to the results for Strategy 1 in Eq. (11), this halves the size of the 0°–270° junction gap at the expense of worsening the 0°–90° junction.

#### 2.A.3 Strategy 3: Minimize the perimeter of the triangle defined by points P_0°_, P_90°_, P_270°_

The triangle in question is illustrated in Fig. 2. The idea is to make the points *P*_0°_, *P*_90°_, and *P*_270°_ as close as possible to one another by minimizing the perimeter of this triangle. In this minimization process it is possible that the triangle collapses into two line segments, which means two points are in the same location, or it could collapse into a point which means all three points are coincident. The minimization problem is to find

(22) $$\begin{array}{cc} perimeter_{\min}=\; & \min_{\left(x,y\right)}\left\{\sqrt{{\left(s+y-x\right)}^{2}+{\left(t-x-y\right)}^{2}}\right.\\ & \;+\sqrt{{\left(u-y-x\right)}^{2}+{\left(v+x-y\right)}^{2}}\\ & \left.\;+\sqrt{{\left(u-s-2y\right)}^{2}+{\left(v-t+2x\right)}^{2}}\right\} \end{array}$$

The minimum of the perimeter function does not have a simple analytical solution but the solution can be determined numerically when *s*,* t*,* u,* and *v* are known.

### 2.B The calibration protocol

Part of this procedure for selecting an origin is specific to the TrueBeam but it could be adapted to other types of medical accelerators.

The electronic portal imaging device (EPID) provided submillimeter resolution and was a good choice to determine jaw positions. The TrueBeam was equipped with a Varian amorphous silicon EPID imager (PortalVision aS1000) which has a pixel pitch of 0.392 mm and has 1024 pixels in the crossplane direction and 768 pixels in the inplane.^13^ Locating the imager to the isocenter plane meant there were no scaling effects to consider. The location of an imaging feature in pixels (*i*,* j*) = (column number, row number) represents the location of the imaging feature in the isocenter coordinate system. The imager has an overall offset between the corner of the imager and the isocenter and the collimator axis is nominally located at (512, 384) when the imager is at position (0, 0, 0). Image features were located with subpixel precision using interpolation techniques. Clews demonstrated 0.17 mm accuracy was possible on an imager with half the resolution of the PortalVision aS1000.^6^

#### 2.B.1 Selection of the origin

The collimator axis was located on the imager using a radio‐opaque marker that was nominally set at the collimator axis. Small errors in the location of this marker from the true collimator axis of rotation do not change the best choice of origin as explained by Fig. 4. A lead crosshair phantom was constructed for this purpose but other marker choices could be made such as a ball bearing embedded in the center of a block tray.^6^ The crosshair phantom consisted of four pieces of 0.5 mm thick lead foil embedded edge‐on into a block tray and arranged to be coincident with the optical crosshairs. The crosshair phantom was imaged at collimator angles 0°, 90°, and 270° and the crosshair's center in the imaging plane were the points *C*_0°_, *C*_90°_, and *C*_270°_. The ideal origin location *P*_0°_ on the imager for each optimization strategy was computed together with the points *P*_90°_ and *P*_270°_.

### 2.C Calibrating the jaws

Once the optimal origin was located, the 1, 19, 5, and 9 cm locations from the origin were computed in pixels using the pixel pitch. The final challenge was to move the linac jaws to these locations during calibration. The TrueBeam's jaw calibration procedure necessitates moving the jaw with the hand pendant thumbwheels by a physical distance measured at the isocenter plane. Varian's method uses the light field to determine the distance on graph paper from the optical crosshair. Since jaw's location relative to the imager could not be seen in real time, the jaw's target position had to be determined in advance. The jaw display could not be used because the display rounds to the nearest millimeter. The primary jaw encoders could be displayed during the calibration procedure and these were ideal for tracking the jaw position. The jaw encoders are part of the control system that track the jaw position with resolvers. It displays up to eight significant figures for which the fourth most significant figure represents position at the millimeter level of precision. Hence the goal was to predetermine what encoder values correspond to the 1, 19, 5, and 9 cm positions and then move the jaws to these encoder values when performing the jaw calibration procedure.

**Figure 5:**
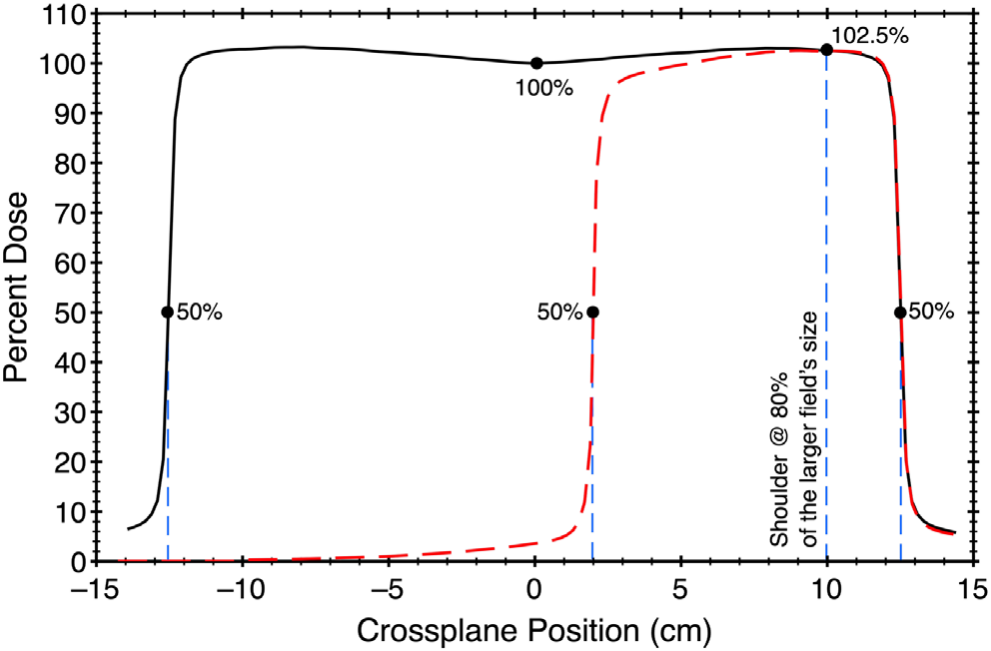
Profiles for large and smaller fields were normalized so their shoulders, defined at 80% of the larger field's size, were matched. The large field was normalized to give 100% to the center and in this example of a 25 × 25 field the shoulder at 10 cm was 102.5%. The smaller asymmetric field was normalized so its shoulder at 10 cm was at also 102.5%. The location of the 50% isodose values define the radiological field sizes.

The encoder values were displayed in service mode and correlated to the jaw position, in pixels, on the imager. Field images were taken and the location of the 50% isodose value was defined to be the position of the jaw. The 50% value was defined relative to the shoulder of the field, located at 80% of the field size. The shoulder of the field was matched to a symmetric field whose center is normalized to 100%. See Fig. 5 for an example.

The graph of encoder value versus pixel value was very linear for both jaws but more so for the X‐jaw than the Y‐jaw. Nonetheless, the residual plot showed an unaccounted pattern in the data variation and a cubic fitting proved to be superior fit. This meant at least four points relating encoder to pixel locations would be required in routine application of this protocol. The 1, 19, 5 and 9 cm positions were converted in to pixel locations and the encoder value were predicted from the cubic equation for these positions. Since the jaw was moved using the hand pendant thumbwheels during the calibration procedure, it was impossible to exactly set the encoder to the desired value. Instead, a range of acceptable encoder values were generated that corresponded to being within 0.1 mm of the desired position. With some patience and practice it was possible to get the jaw position within the 0.1 mm window using the hand pendant.

Once the calibration protocol was completed, the field size accuracy over the entire range of motion was verified by using both radiological and optical measurements and the junctions were verified.

## 3 RESULTS

### 3.A Evaluating the junction

There are a number of ways one can evaluate a junction. The EPID imager together with in‐house software was used. This software is a fast and effective tool for evaluating the junction. The program adds four quarter‐blocked field images and takes profiles through the four junctions. The images are a mixture of quarter blocked fields with collimator angles 0°, 90°, and 270° that combine to give each junction pair X1:X2, Y1:Y2, X1:Y2,…. It was recognized that dosimetric profiles taken from the imager were not accurate representations of the dose profiles.^6^ The EPID imager exaggerated the dosimetric valleys or peaks at a junction by a multiplicative factor of 1.4 in comparison to the same junction taken with a film. The QA action and tolerance levels were adjusted to account for this factor. Table 1 shows the junctions before and after the application of this jaw calibration protocol.

**Table 1:** The TrueBeam junctions before and after the calibration procedure. An EPID took four quarter‐blocked fields images that in house‐software sums and takes four profiles. The EPID measurement tends to over/underestimate the junction by a factor of 1.4 in comparison to the junction measured with an ion chamber or with film

Jaw & collimator junction pairs	Peak max/valley min prior to calibration	Peak max/valley min post to calibration
X1 C0°–X2 C0°	−23.1%	−2.5%
Y1 C0°–X2 C90°	−24.7%	3.9%
X2 C0°–Y2 C90°	−7.7%	13.6%
Y1 C0°–Y2 C0°	−17.9%	5.6%
X1 C0°–Y1 C90°	−33.4%	5.3%
Y1 C0°–X1 C270°	−24.2%	5.6%
X2 C0°–Y1 C270°	−26.8%	−8.3%
Y2 C0°–X1 C90°	−18.9%	−4.1%

### 3.B Selecting the origin

**Figure 6:**
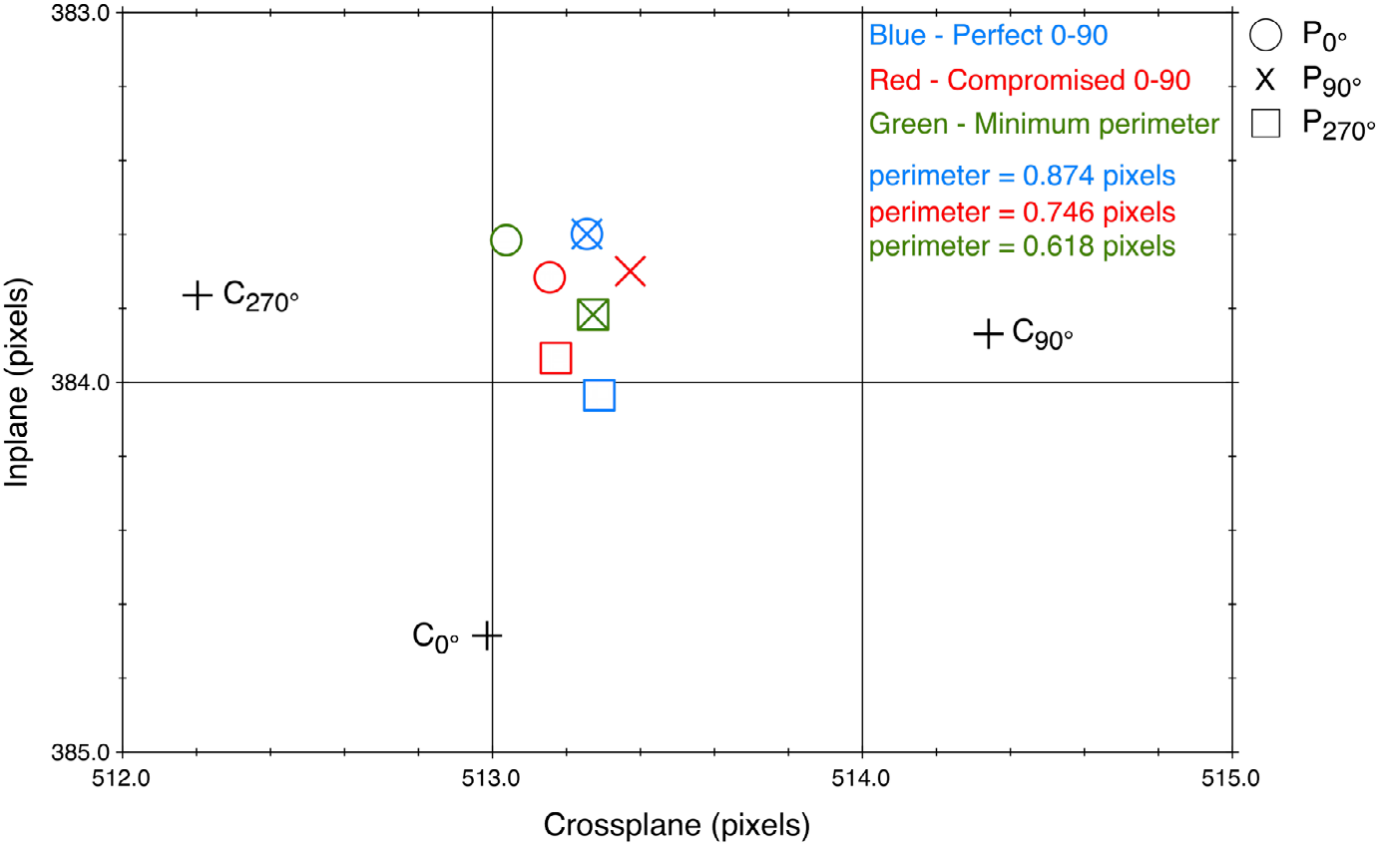
The crosshair locations *C* for collimator angles 0°, 90° and 270° determined where the ideal origin choice was. Three strategies for selecting the origin were: (a) (Blue) The origin *P* was computed to produce a perfect geometric match for the collimator 0°–collimator 90° junction; (b) (Red) Compromise the perfect junction 0°–90° junction to improve the 0°–270° junction; and (c) (Green) minimize the perimeter distance |*P*_0°_–*P*_90°_| + |*P*_0°_–*P*_270°_| + |*P*_90°_–*P*_270°_|.

Figure 6 shows an example of the optimal origin choice for each of the three presented strategies. The crosshair phantom showed *C*_0°_ shifts by about 1.25 pixels (0.49 mm) to either *C*_90°_ and *C*_270°_. In clinical, TG142 and CPQR allow the machine crosshair have < 1 mm radius walkout. The optimal origin choice shows much smaller shifts of the order of 0.2–0.4 pixels (0.078–0.157 mm) between *P*_0°_, *P*_90°_, and *P*_270°_. The three strategies had perimeters 0.87 pixels (0.341 mm), 0.75 pixels (0.294 mm), and 0.62 pixels (0.243 mm) for the first, second, and third strategies respectively. We examined three TrueBeams and at least three different measurements each TrueBeam for more than 6 months. The differences in optimal origin location between the three strategies were small (of the order of 0.2 pixel <0.1 mm). It is technically challenging to move the jaws in the calibration process less than 0.1 mm and show different effects of these three strategies.

### 3.C Calibrating the primary encoder

**Figure 7:**
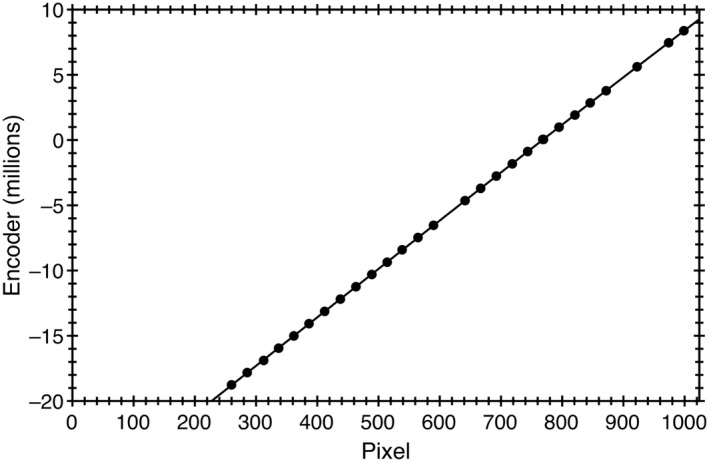
The encoder versus pixel value for the Y1 jaw. A cubic fit provides a better prediction for the encoder values.

With the origin selected, the pixel locations of the 1, 19, 5, and 9 cm jaw positions were now known. It remained to relate the encoder value to these pixel values. Figure 7 plots an example this relationship for the Y1 jaw with similar results for X1, X2, and Y2.

## 4 DISCUSSION

This method uses the TrueBeam's EPID imager but it lends itself to extensions beyond the TrueBeam and the imaging system. The key properties for a successful system are (a) relating the fixed imaging coordinate system to the collimator coordinate system with sufficient precision and (b) relating the jaw position to these coordinate systems. While the technical details may differ, the general principles will be the same.

Numerous refinements to the theory could be addressed. Two involve the imager's position and orientation. The imager was assumed to be located on the isocenter and have no inclination relative to the isocenter plane. This was not necessarily true. An imager further away or closer to the source would have a scaling factor. The pixel pitch is the physical size of the imaging elements but each pixel when projected back to the imaging plane on the isocenter will increase or decrease in size via this factor. The imager may also be tilted relative to the imaging plane. This inclination will introduce an image distortion along inplane that increases or decreases crossplane lengths due to changing scales and uniformly changes inplane lengths due to the inclination of the imager relative to the imaging plane. The above factors will cause geometric distortions in the acquired images. While the imager's technical specifications and performance were sufficient to give clinically acceptable jaw calibration results, it would be useful to quantify these effects and correct for them in the calibration procedure if needed.

The in‐house software assumes the four quarter blocked fields were set squarely to each other but this may not be true. The collimator rotation has tolerances and display rounding issues on the order of 0.1°. There can be junction quality issues if the collimator is not set perfectly at 0°, 90°, and/or 270°.

It was noticed the third strategy appeared to have a solution where both *P*_90°_ and *P*_270°_ were located midway on the line between *C*_90°_ and *C*_270°_. Speculating this may be true for many practical measurements, a closer look at the mathematical details was warranted. These two points are equal if

(23) $$x=\frac{t-v}{2}$$

(24) $$y=\frac{u-s}{2}$$

and the minimum perimeter is

(25) $$perimeter_{\min}=2\left\{\sqrt{{\left(\frac{s-t+u+v}{2}\right)}^{2}+{\left(\frac{s+t+v-u}{2}\right)}^{2}}\right\}$$

**Figure 8:**
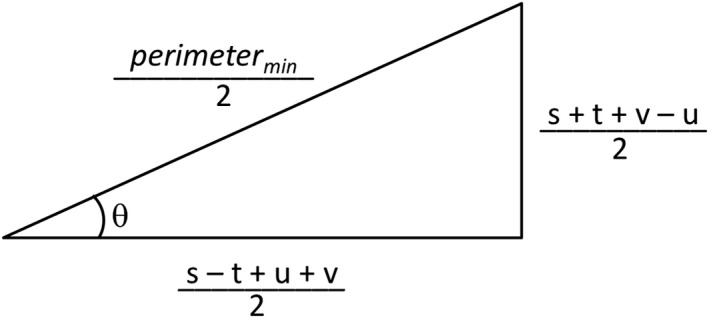
The proposed solution for the minimum perimeter can be visualized geometrically in terms of the collimator walkout parameters *s*,* t*,* u* and *v,* and an angle *θ*.

This proposed solution can be represented geometrically as a right triangle with angle *θ*, as shown in Fig. 8.

A first order asymptotic expansion with small parameters α and β around this proposed solution was made to confirm if this was a local minimum. Setting

(26) $$x=\frac{t-v}{2}+\alpha$$

(27) $$y=\frac{u-s}{2}+\beta$$

the perimeter function has the asymptotic expansion

(28) $$perimeter=perimeter_{\min}-2\left(\alpha \cos\theta +\beta \sin\theta \right)+O\left(\alpha^{2},\beta^{2},\alpha \beta \right)$$

The value *perimeter*_min_ is a local minimum provided

(29) $$\frac{\alpha }{\beta }<-tan\theta$$

If tan*θ* <0, then with relatively weak restrictions on α and β Eq. (29) is true. However, the same cannot be said when tan*θ* > 0 because the ratio of α and β can be positive or negative and the proposed solution behaves more like a saddle point than a local minimum.

## 5 CONCLUSIONS

A jaw calibration protocol using the EPID on the TrueBeam linear accelerator was developed that can be adapted to other accelerators. The imager has sufficient resolution for determining jaw positions to submillimeter accuracy and precision. The technique was capable of producing junctions that can be within ±5% dosimetric homogeneity.

As there is installation error between the TrueBeam crosshair and the collimator axis of rotation, this crosshair is not necessary a good choice as origin for the jaw calibration to achieve good junctions. Our mathematical model finds the optimal origin by analyzing the measurement results from EPID images.

Multiple strategies exist for the selecting an origin using our model and each strategy presents pros and cons. The perfect match for collimator 0°–90° is possible but at the expense of the collimator 0°–270° junction. Nevertheless this can be the ideal choice because a field with collimator set to 270° can be duplicated with the collimator at 90°, eliminating the need to use 270° collimator angles clinically.

Our long‐term observations on three TrueBeams indicate that these three methods of picking origin have very small differences. This means the TrueBeam's collimator walkout is small and stable and the optimal origins picked by the three strategies of our model are basically very close to the collimator axis of rotation.

Our mathematical model and the method of using EPID imaging to determine the rotation walkout can also be used on treatment couch and gantry walkout studies. However, the calibration (installation) procedures will not be as easy as jaw calibration. The gantry walkout study with EPID imager will be more complicated with sag issue.

The validity of this procedure was confirmed on three TrueBeams. It has taken them from having clinically unacceptable junctions to dosimetrically uniform junctions while maintaining the jaw accuracy over the full dynamic range of motion.

## CONFLICT OF INTERESTS

There are no conflict of interests.
